# Optical Coherence Tomography Angiography of the Choriocapillaris in Subclinical Toxoplasma Chorioretinitis: A Case Report

**DOI:** 10.7759/cureus.41543

**Published:** 2023-07-07

**Authors:** Ahmed B Alsatrawi

**Affiliations:** 1 Ophthalmology, Salmaniya Medical Complex, Manama, BHR

**Keywords:** juvenile idiopathic arthritis, infectious uveitis, toxoplasma gondii, ocular toxoplasmosis, retina, uveitis, octa, ophthalmologic findings of ocular toxoplasmosis, oct angiography, oct (optical coherence tomography)

## Abstract

This report describes the optical coherence tomography angiography (OCTA) findings detected at the choriocapillaris slab before the clinical picture of acute toxoplasmosis choroiretinitis (TCR) by following up a patient with serial images of OCT and OCTA from the quiescent to the active stage of the disease. In this case, the increased thickness of the choroid in the OCT B-scan and the prominent flow void at the choriocapillaris slab of the OCTA were detected early in the course of the disease. OCTA is a useful imaging technique in TCR and might help in predicting the TCR lesion at the subclinical stage.

## Introduction

Toxoplasmosis is one of the most common causes of infectious uveitis worldwide. [1]. It is caused by an obligate, intracellular protozoan parasite named *Toxoplasma gondii* that tends to affect all warm-blooded vertebrates including humans [1,2]. Despite toxoplasmosis being described to have a wide spectrum of presentation within the eye, the condition is commonly labeled as a typical "ocular toxoplasmosis" (OT) when a large necrotizing chorioretinitis lesion is present at the posterior pole with a variable degree of vitreous inflammation [3]. The diagnosis of OT is usually based on this clinical picture with a positive serology to the parasite antibodies [3].

The rapid advances in retinal imaging technologies made them more prominent in diagnosing, treating, and monitoring patients with posterior uveitis. Optical coherence tomography angiography (OCTA) is one of these technologies. It's a comparatively new imaging modality introduced less than 10 years back as an evolvement from optical coherence tomography (OCT), the technology of the 1990s that revolutionized the diagnosis and management of retinal diseases. OCTA combines both the structural findings seen on OCT and the functional study of blood flow [4-6].

In this case, we report changes seen on OCTA images of the choriocapillaris (CC) slabs of a young patient with recurrent OT. The aim and emphasis were to highlight the early vascular changes at the level of the CC detected before the manifestations of clinical evidence and diagnosis of reactivated toxoplasma chorioretinitis (TCR).

## Case presentation

**Figure 1:**
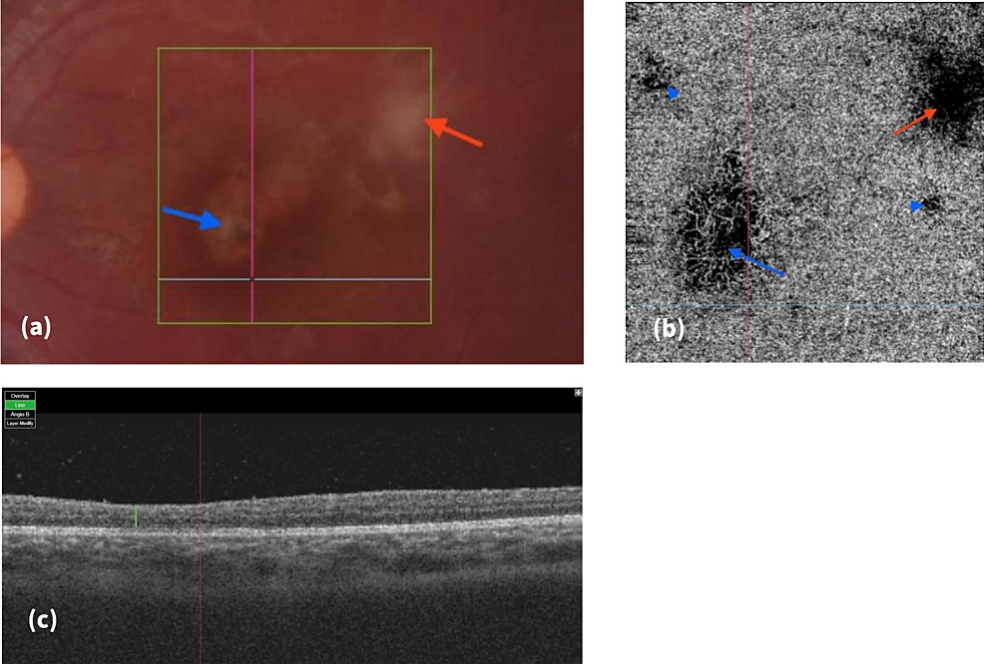
Right eye color fundus photo, en-face OCTA of the choriocapillaris, and OCT B-scan in February 2022. (a) Color fundus photo of the primary inactive TCR scar (blue arrow) and the secondary active TRC (red arrow); (b) en-face OCTA (4.5 X 4.5 mm) at the choriocapillaris, restored vascular network at the flow void area of the primary TCR (blue arrow), multiple satellite flow void areas (blue arrowheads) and a prominent flow void with vascular distortion at the site of the secondary TCR; (c) OCT B-scan (corresponding to the blue horizontal line in (a) and (b)) showing thin retina (green line). OCTA: optical coherence tomography angiography; OCT: optical coherence tomography; TCR: toxoplasma chorioretinitis

This is a case of a nine-year-old female with juvenile idiopathic arthritis (JIA) on methotrexate with a history of treated acquired TCR before the onset of JIA. The patient had regular follow-ups in the uveitis clinic due to the history of JIA, TCR, and the long-term use of immunosuppressive therapy. On February 2022, the patient was found to have a reactivation of her OT (Figure 1), and anti-toxoplasmosis therapy (sulfamethoxazole and trimethoprim) was initiated with close follow-ups.

**Figure 2:**
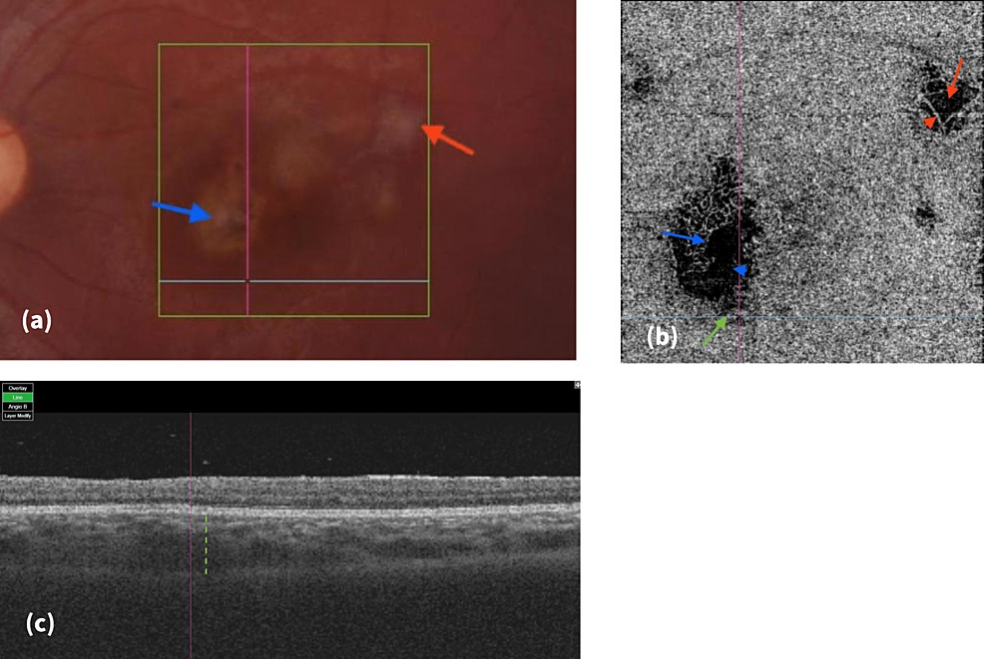
Right eye color fundus photo, en-face OCT-A of the choriocapillaris, and OCT B-scan in April 2022. (a) Color fundus photo, the primary TCR scar (blue arrow) and improving secondary TCR (red arrow); (b) En-face OCTA (4.5 X 4.5 mm) at the choriocapillaris, new vascular distortion (blue arrowhead), reduced size of the old restored vascular network (blue arrow), inferior enlargement of the flow void area of the primary TCR (green arrow), and improved flow with vascular restoration of the secondary TCR (red arrow and arrowhead); (c) OCT B-scan (corresponding to the blue horizontal line in (a) and (b)) showing the increased thickness of the choroid (dotted green line). OCTA: optical coherence tomography angiography; OCT: optical coherence tomography; TCR: toxoplasma chorioretinitis

**Figure 3:**
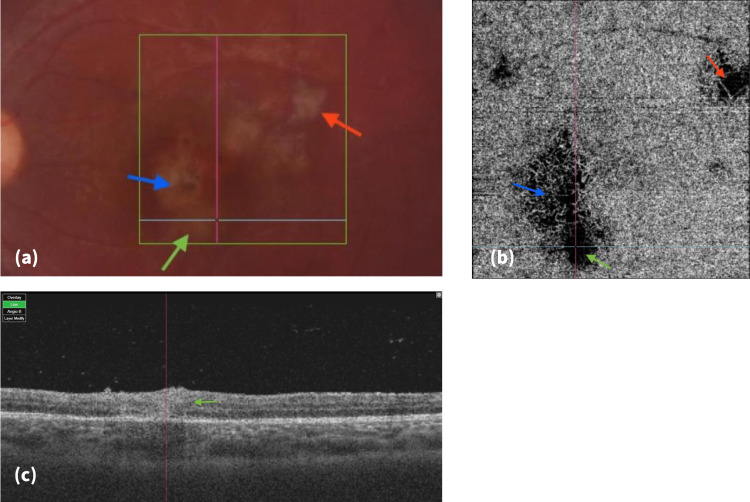
Right eye color fudus photo, en-face OCTA of the choriocapillaris, and OCT B-scan two weeks later, on third follow-up. (a) Color fundus photo, the primary TCR (blue arrow), secondary TCR (red arrow), and new active TCR (green arrow); (b) En-face OCTA (4.5 X 4.5 mm) of the choriocapillaris, prominent flow void inferiorly (green arrow) extending from the old primary TCR (blue arrow), and further flow improvement in the secondary TCR (red arrow); (c) OCT B-scan (corresponding to the blue horizontal line in (a) and (b)) showing disorganized, thickened, and hyper-reflective retina layers. OCTA: optical coherence tomography angiography; OCT: optical coherence tomography; TCR: toxoplasma chorioretinitis

Two months later, the secondary active TCR showed obvious improvement (Figure 2). However, on the same follow-up visit, the OCTA images of the CC slab showed an obvious inferior enlargement of the flow void area of the old primary inactive TCR scar associated with the destruction of the nearby vascular network (Figure 2). Two weeks later, on her next follow-up, the clinical examination, fundus photo, OCT, and OCTA images all revealed and supported the diagnosis of a new active lesion of TCR overlapping the site of the inferior flow void area seen two weeks earlier on the CC slab of the OCTA (Figure 3).

## Discussion

With their favorable visualization and image quality through media opacities, swept-source OCT and OCTA technologies are becoming popular as valuable tools in diagnosing, treating, and monitoring patients with posterior uveitis [7]. TCR is one of these entities, with many previous reports describing both the acute active lesion and the old quiescent scar [8-10]. Going through all the findings reported previously is beyond the spectrum of this report; however, the vascular changes observed at the level of the CC in the OCTA are of interest and relevance. In acute active TCR, the CC slab shows vascular obliteration and reduced blood flow while later in the course of the disease, at the quiescent stage, we see partial restoration of these vascular networks with improvement in the overall blood flow [9,10].

A study by Zicarelli et al. in 2021 supported the idea that these areas of reduced flow in the CC are actual and not a shadowing artifact from the retina [10]. Their study also supported the essential role played by the choroid in the pathophysiology of TCR and its wider involvement as compared to the retina, confirmed by OCTA.

To the best of our knowledge, no previous reports described the OCT and OCTA changes before the clinical diagnosis of active TCR. Due to the young age of our patient and the long-term use of immunosuppressive therapy, we followed the case closely with a series of OCT and OCTA images. The new enlargement of the flow void area seen inferior to the quiescent TCR (Figure 2b) was initially thought to be an incidental finding or some process of vascular remodeling and could not be related to a significant change in the clinical exam or other imaging scans, except for the increased choroidal thickness seen on OCT B-scan (Figure 2c).

The two-week follow-up examination revealed typical findings of a new active TCR at the site of flow void enlargement seen earlier in the CC slab (Figure 3). These early choroidal changes, whether primarily due to parasitic invasion or secondary to inflammatory reaction represented an important early stage in the pathophysiology of TCR. It further supported the idea that the choroidal involvement in TCR is not only larger than what can be seen but earlier too, proceeding other obvious clinical features.

## Conclusions

In this paper we highlighted the importance of choroidal imaging using OCT and OCTA in patients with TCR. Imaging this layer has great value in both the clinical and subclinical stages of the disease. Increased choroidal thickness in the OCT and the associated flow voids in CC slabs of the OCTA both were detected subclinically in our patient. Further series on the subject are needed to illustrate more and in detail these subclinical findings of TCR on OCT/OCTA and how they can be utilized to predict and follow the disease activity, especially in patients with compromised immune systems.
